# O.4.4-4 SPORTLAND HESSEN bewegt as an innovative health enhancing physical activity program at state level based on a Health in All Policies (HiAP) approach

**DOI:** 10.1093/eurpub/ckad133.201

**Published:** 2023-09-11

**Authors:** Béatrice Frank, Lena Ondrasch

**Affiliations:** Hessische Arbeitsgemeinschaft für Gesundheitsförderung e. V. (HAGE), Germany; beatrice.frank@hage.de; Hessische Arbeitsgemeinschaft für Gesundheitsförderung e. V. (HAGE), Germany; beatrice.frank@hage.de

## Abstract

**Purpose:**

The lack of physical activity in our daily lives and the associated health consequences are also a far-reaching health problem in Hesse, which has been amplified by the corona pandemic. To counteract this, Hesse set up its state program “SPORTLAND HESSEN bewegt”.

The aim is to offer the Hessian population a more diverse and better coordinated range of physical activity promotion measures across all ages. This includes raising awareness of the multiple benefits of promoting physical activity, increasing the intersectoral cooperation between all relevant actors, linking the numerous existing efforts to promote physical activity in profitable manner and expanding programs and structures across all ages.

The state program, which sees its mission as a challenge for society as a whole, is primarily targeting all relevant multipliers who can make Hessen more active: from politics to providers of physical activity programs in the municipalities. Not only sports organizations play a central role here, but also players from the fields of education, urban development, transport, health, environment and many more.

**Project description:**

To this date five state ministries, the state sports association as well as the state association for health promotion are involved as well as many other partners.

For this purpose, a structure was created that enables all stakeholders to work together, taking into account the different age groups. As think tanks for the development of measures to promote physical activity, interministerial and intersectoral working groups have been initiated. In addition, coordinators for the promotion of physical activity at municipal level are being established, funding programs for sport clubs were set up and exercise checks are being carried out at primary schools.

**Conclusions:**

As the WHO states in their global action plan on physical activity, increasing physical activity requires a systems-based approach. However, there is no blueprint for such a HiAP approach for the promotion of physical activity.

State government, organized sport and many other partners are pulling together to motivate citizens to adopt a more active lifestyle. By working together, the stakeholders and citizens will hopefully benefit of the synergies and further innovative measures and approaches can be established.

